# HLA-DQB1*02, HLA-DRB1*03 alleles and HLA-DQB1*02-HLA-DRB1*03 haplotype confer a significant risk of celiac disease in the Mauritanian population

**DOI:** 10.1186/s12876-026-05086-9

**Published:** 2026-07-16

**Authors:** Saleck Mahfoudh Ebebecar, Malak Salame, MohamedHameyin, Tijani Hamed, Fatimetou Vetten, Tetou Soumbara, Mohamed lemine Hmeity, Abdallahi Sidi Mohmed, Houria Abdelghaffar, Ahmed Houmeida

**Affiliations:** 1 Research Unit on Biomarkers in the Mauritanian Population (URBPM), University of Nouakchott, Nouakchott, Mauritania; 2 Laboratory of Biotechnology, Agro-Food, Materials and Environment (LBAME), Faculty of science and technology Mohammedia, Hassan II university of Casablanca, BP 146, 20650 Mohammedia, Morocco; 3 National Institute of Hepato-Virology (INHV), Nouakchott, Mauritania

**Keywords:** HLA, Celiac disease, Gluten, Mauritania

## Abstract

**Background and aim:**

Celiac disease (CD) is an autoimmune disorder primarily caused by an abnormal immune response to dietary gluten in human leucocytes class II antigens carriers.

This preliminary study aimed to assess the specific risk conferred by DQB1 and DRB1 alleles to celiac disease development in the Mauritanian population.

**Materials and methods:**

Frequencies of HLA-DQB1and HLA-DRB1alleles and haplotypes were evaluated in 50 celiac disease (CD) patients. The association of HLA-DQB1 and HLA-DRB1with the disease was tested using a control group of 50 non-CD healthy individuals.

Genotyping was performed by PCR-SSP and next-generation sequencing (NGS).

**Results:**

Genotyping of the studied population revealed that the most frequent alleles of DQB1/DRB1gens among CD patients were DQB1*02(70%) and DQB1*03(9%), DRB1*03 (52%) was the largest group followed by DRB1*07 (12%).

The major allelic combination was DRB1*03–DQB1*02, with a frequency of 47%.

Analysis of allele frequency in patients and controls revealed a significant association between CD and the two key HLA alleles here respectively DQB1*02(χ^2^ = 44.46; *p* = 2.59 × 10⁻^11^) and DRB1*03 (χ^2^ = 8.32; *p* = 3.9 × 10⁻^3^). Significant association was also found with DQB1*02-DRB1*03 (χ^2^ = 31.47; *p* = 2.0 × 10⁻⁸). However, no considerable risk of the disease was conferred by any of the other haplotypes tested here.

**Conclusion:**

These preliminary results, consistent with previous literature on CD genetic susceptibility, support the predominant role of DQB1*02 and DRB1*03 alleles and haplotypes in celiac onset in our population. The availability of NGS typing for celiac disease maybe an additive tool to exclude symptoms ambiguity during CD diagnosis in our medical facilities.

## Introduction

Human leukocyte antigens (HLA) have been closely related to the natural history of numerous diseases particularly through its implication in the body's immune response [1, 2]. Gluten is a group of proteins found in wheat, barley or rye and important for the shape and plasticity of various foods [3]. Most of people can eat food with gluten without any side effect, however, in some people, gluten can trigger a range of health issues of which celiac disease (CD) is the most known [4]. This condition may cause damage to gut (small intestine) with a set of symptoms such as diarrhea, abdominal pain and bloating [3]. CD is classified as a chronic autoimmune disorder provoked by an immune response to gluten ingestion in genetically predisposed individuals [5]. Indeed, confirmation of the disease symptoms is worldwide implemented, according to NASPGHAN(North American Society for Pediatric Gastroenterology, Hepatology and Nutrition), by a titer 10 times the upper limit of IgA tissue transglutaminase antibody (IgA-TTG) followed by genetic testing for HLA class II molecules [6]. For instance, approximately 95% of Caucasians with CD express HLA-DQ2 (HLA-DQA1*05; HLA-DQB1*02) haplotype, the remaining 5% of patients possess either the HLA-DQ8 haplotype (HLA-DQA1*03; HLA-DQB1*03/) or another variant of HLA-DQ2 [7, 8]. Although less explored in North African and sub-Saharan CD patients, the allele and haplotype of HLA antigens showed similar genetic pattern with different frequencies [9].

Celiac disease was also strongly associated with the HLA class II DR markers with evidences of for a genetic heterogeneity of this region reported in different populations [10].

Although a HLA typing showing the absence of susceptibility alleles may help exclude the diagnosis of celiac disease, the presence of these alleles cannot be used as a diagnostic tool, since in the general population, about 30–40% of individuals carry these susceptibility alleles, while only about 3–4% of them will actually develop celiac disease [11]. We have previously assessed the global and ethnic distribution of the main HLA class I and class II antigens in the Mauritanian population [12]. Here, we investigated the potential risk conferred by DQB1 and -DRB1 alleles and haplotypes to CD onset in our population.

## Materials and methods

### Target population

HLA-DQB1 and HLA-DRB1 allele frequencies were explored in 50 celiac disease patients recruited at the National Institute of Hepato-Virology (INHV) and the pediatric department of CHIVA Clinic respectively by a gastroenterologist and a pediatrician based on CD symptoms, rising serum levels of anti-tissue transglutaminase (anti-tTG IgA) and/or intestinal biopsy findings. Association between DQB1 and DRB1alleles and haplotypes with CD was assessed by contrast with a group of 50 healthy non-CDindividuals.

### DNA Extraction

Genomic DNA extraction was performed from whole blood samples collected in EDTA tubes from each individual in the target population, following the protocol provided by the Qiagen kit (QIAamp DNA Mini Kit, Cat. No. 51104). Extracted DNA was evaluated quantitatively using a Qubit fluorometer and qualitatively by agarose gel electrophoresis.

### HLA-DQB1/DRB1Genotyping

HLA-DQB1/DRB1 loci typing in CD patients and healthy were carried out primarily using PCR–SSP (Polymerase Chain Reaction – Sequence Specific Primers) Lambda kits and HLA Fusion™ software, version 3.0.0 for allele identification.

To further confirm PCR–SSP data, genotyping was cross-validated in 24 CD patients by high-resolution. next-generation sequencing (NGS), using Nanopore MinIon sequencer with Nanotype kits.Amplification, library preparation and sequencing were performed according to the sequencing kit procedure. Computational analysis was carried by Omixon software compatible with nanopore technology.

To confirm analogy of both HLA typing here, 2 CD patients and controls were HLA typed using PCR-SSP and nanopore sequencing

### Statistical analysis

The prevalence and association of HLA -DQB1/DRB1 alleles and haplotypes with celiac disease was analyzed by SPSS ver25.0(SPSS In Chicago, IL, USA).

Allelic frequencies (Fr %) were calculated as: Fr (%) = (n allele/N total) X 100 with, n is the count of the given allele and N the total number of alleles analyzed in the group.

## Results

### Prevalence of HLA-DQB1and HLA-DRB1 alleles and haplotypes in celiac disease patients

In this cohort, the most frequent alleles among CD patients were DQB1*02 (70%), DQB1*03 (9%) at DQB1 locus, DRB1*03 (52%) and DRB1*07(12%) at the DRB1 locus respectively (Table 1). Calculation of HLA-DQB1-DRB1 locus combinations showed that the majority of haplotype carriers were DQB1*02-DRB1*03 (47%), followed by DQB1*02 -DRB1*07 (10%), DQB1*02 -DRB1*01 (8%) and DRB1*03–DQB1*03 (3%) (Table 1).

**Table 1:** Distribution of HLA-DQB1/DRB1 alleles and haplotypes HLA-DQB1-DRB1 among celiac patients

HLA-DQB1Patients:(n=100)			HLA-DRB1Patients:(n=100)			haplotypes frequences		
Allele	Nb	Fr %	Allele	Nb	Fr %	haplotype	Nb	Fr %
DQB1*02	70	70%	DRB1*03	52	52%	DRB1*03‐ DQB1*02	47	47%
DQB1*05	09	09%	DRB1*07	12	12%	DRB1*07-DQB1*02	10	10%
DQB1*03	09	09%	DRB1*01	9	9%	DRB1*01-DQB1*02	08	08%
DQB1*06	09	09%	DRB1*13	6	6%	DRB1*03-DQB1*03	03	03%
DQB1*04	01	01%	DRB1*04	4	4%	DRB1*11-DQB1*02	03	03%

*n* Number of alleles, *Nb* Number of repetitions for each allele, *FR* frequency

### Association of HLA-DQB1 and HLA-DRB1 alleles and haplotypes with celiac disease

Reviewing their role as risk or protective factor for CD, the distribution of HLA-DQB1 alleles among patients and controls showed a significant association between DQB1*02 (χ^2^ = 44.46, *p* = 2.59 × 10⁻^11^; OR = 8.27), DQB1*03 (χ^2^ = 19.38; *p* = 1.07 × 10⁻^5^; OR = 0.18), DQB1*04 (OR ≈ 0.12; *p* < 0.05) alleles and the disease. In contrast, no association was observed for DQB1*05 (OR ≈ 0,45; *p* =0,098) and DQB1*06 (OR ≈ 0,52%; *p* =0,200) (Table 2). The analysis revealed also a significant association between DRB1*03 and celiac disease (χ^2^ = 8,32; *p* = 3,9 × 10⁻^3^; OR = 3.48) and DRB1*01 (OR ≈ 4.85 *p*= 3,0 × 10⁻^2^).However, no significant association was observed for DRB1*07 (χ^2^ = 0,89 *p*= 3,45 × 10⁻^1^ OR=1,57)and DRB1*04 (χ^2^ = 0, 00 *p*=1,0, OR=1,00)(Table 3).

**Table 2:** Distribution of HLA-DQB1 alleles and statistical tests (χ^2^, *p*-value, and odds ratio) among celiac patients and controls

HLA-DQB1	*Patients:(n=100)*		*Controls* (*n*=100)		X^2^	*P*-value	OR	IC 95%
	Nb	Fr %	Nb	Fr %				
DQB1*02	70	70%	22	22%	44,46	2,59 × 10^−11^	8,27	4,37 −15,65
DQB1*05	09	09%	18	18%	2,74	0,098	0,45	0,19 - 1,06
DQB1*03	09	09%	36	36%	19,38	1,07 × 10^−5^	0,18	0,08 - 0,39
DQB1*06	09	09%	16	16%	1,65	0,200	0,52	0,22 - 1,24
DQB1*04	01	01%	07	07%	3,26	0,071	0,13	0,02 - 1,11

*n* Number of alleles, *Nb* Number of repetitions for each allele, *OR* odds ratio, *X*^*2*^ Chi-squared test, *IC* confidence interval

**Table 3:** Distribution of DRB1 locus alleles and statistical tests (χ^2^, *p*-value, and odds ratio) among celiac patients and controls

*HLA-DRB1*	*Patients:*(*n*=100)		*Controls* (*n*=100)		*X*^*2*^	*P-value*	*OR*	*IC 95%*
	*Nb*	*Fr %*	*Nb*	*Fr %*				
DRB1*03	52	52%	24	24%	8.32	3,9 × 10^−3^	3,43	1,88–6,28
DRB1*07	12	12%	08	08%	0.89	3,45 × 10^−1^	1,57	0,61–4,02
DRB1*01	09	09%	02	02%	4.71	3,0 × 10^−2^	4,85	1,02–23,1
DRB1*13	06	06%	27	27%	16	6,0 × 10^−5^	0,173	0,068 −0,441
DRB1*04	04	04%	04	4%	0. 00	1,0 × 10^0^	1,00	0,24–4,09

*n* A total number ofalleles, *Nb* Number of repetitions for each allele, *OR* odds ratio, *X*^*2*^ Chi-squared test, *IC* confidence interval

Assessment of CD risk by HLA-DQB1 -HLA-DRB1allele combinations (Table 4) showed that the most prominent haplotype DQB1*02 -DRB1*03 (47%) was significantly associated with celiac disease (X^2^ = 31.47 *P*-value =2.0 × 10⁻⁸, OR = 7.17). In contrast, none of the other present haplotypes was found affecting the disease development.

**Table 4:** Distribution of HLA-DQB1-DRB1 in celiac patients haplotypes and controls

*HLA-DQB1-DRB1*	*Patients(n=100)*		*Control (n=100)*		*X*^*2*^	*P-value*	*OR*	*95% IC*
	*Nb*	*Fr %*	*Nb*	*Fr %*				
DRB1*03‐ DQB1*02	47	47%	11	11%	31.47	2,0 × 10^−8^	7,17	3,34 - 4,31
DRB1*07-DQB1*02	10	10%	04	4%	2,76	9,6 × 10^−2^	2,67	0,79 - 7,79
DRB1*01-DQB1*02	08	08%	0	0%	8,33	3,9 × 10^−3^	∞(brut)	1,05–324,51
DRB1*03-DQB1*03	03	03%	10	10%	4,03	4,5 × 10^−2^	0,28	0,09 - 1,07
DRB1*11-DQB1*02	03	03%	03	03%	0,827	1,0 × 10^0^	1,00	0,22 - 4,52

*n* A total number of alleles, *Nb* Number of repetitions for each allele, *OR* odds ratio, *X*^*2*^ Chi-squared test, *IC* confidence interval

Univariate logistic regression analysis revealed a highly significant association between celiac disease and the presence of the HLA-DQB1*02 allele (SE = 0.59, Waldχ^2^ = 19.84, *p* < 0.001), the HLA-DRB1*03 allele (SE = 1.934, Wald χ^2^ = 16.35, *p* < 0.001), as well as the DQB1*02–DRB1*03 haplotype (Table 5).

**Table 5:** Univariate logistic regression analysis of HLA- DQB1_02 and HLA- DRB1_03 alleles and DRB1*03–DQB1*02 haplotype combination associated with Celiac disease status

Predictor	B	SE	Wald X2	OR (95% CI)	*P*-value
DQB1_02	2.63	0.59	19.84	14 (4.38–44.7)	< 0.001
DRB1_03	1.934	0.47	16.35	6.92(2.71–17.67)	< 0.001
Haplotype_Combo (DRB1*03–DQB1*02)	2.45	0.47	26.49	11.69(4.58–29.82)	0.00

## Discussion

HLA class II antigens have been specifically correlated with triggering several auto-immune diseases although the frequency of these disorders cannot be explained by genetics alone [13].

In this pilot study, we have used the highly accurate and affordable NGS Nanopore technology [14] to assess, in our population, the prevalence and conferred risk of two prominent HLA class II genes associated with celiac disease onset. Indeed, among the fifty CD patients tested, a good number (70%) carried DQB1*02 allele followed by DQB1*03 (9%). DRB1*03 was also found in 52% of patients while 12% carried DRB1*07. As a result, we have showed that 79% of CD patients were DQB1*02 orDQB1*03 and 64% carried either DRB1*03 or DRB1*07. Besides, a significant association with celiac disease was observed for the two main alleles respectively HLA-DQB1*02(*p* < 3×10⁻^11^) and HLA-DRB1* 03 (*p* < 0.004). Furthermore, the most frequent haplotype DQB1*02-DRB1*03 (47%) was also significantly associated with the disease (*p* =2.0 × 10⁻⁸) in our cohort.

Although preliminary, these results underlined the major role of DQB1*02 andDRB1*03in genetic susceptibility to celiac disease in our population as reported by numerous reports worldwide [15]. For instance, of the sixty pediatric patients with CD typed at the University Clinical Centre of Kosovo (UCCK), the three most frequent alleles were HLA – DQB1*02 (58%), HLA-DRB1∗03 (38.3%), DRB1∗07 (17.5%) which also presented a much higher frequency among CD patients compared to control group (*p*< 0 0001, *p*< 0 0001 and p = 0 0023, respectively) [16]. In the Iranian population, a study performed in as similar size cohort revealed that among 40 CD individuals DQB1*02 was present in 33.75% of patients versus only 8.75% of controls with, overall, 65% of the patients affected by celiac disease hostingDQB1*02 or DQB1*03 alleles [17]. Likewise, 38 out of 49 (77.5%) Syrian children with CD were reported to carry either of these two main alleles [18]. Using PCR-based amplification of HLA and NGS sequencing, as in our protocol, on143Scandinavian CD patients and 135 non-celiac disease controls, showed thatDQB1*02-DRB1*03 haplotypes conferred a strong risk of celiac disease (RR = 6.34; *P*-value <.0001) [19]. A study conducted on the modifying effect of HLA haplotypes located trans to DQB1*02-DRB1*03 in celiac patients of Southern Europe also found that homozygous DQB1*02-DRB1*03/DQB1*02-DRB1*03 and heterozygousDQB1*02-DRB1*03/DQB1*02-DRB1*07 were the only combinations that showed a significant and independent association to CD. That study proposed a gene dosage effect of the DQB1*02-DRB1*03 haplotype [19, 20].

In North Africa, although the disease frequency has been fast increasing, genetic etiology of the disease was rarely investigated. However, a study using HLA class II (HLA-DQA1/DQB1/DRB1) typing in Moroccan CD patients compared to a control group from has strengthened the risk gradient for CD in the Moroccan population [9].

In the Tunisian population, the risk of developing celiac disease was also estimated to be 6.8 times greater for those having a double dose of DQB1*02 [21].

Although our work was centered primarily on the potential risk of CD related to HLA DQB1*02 and HLA-DRB1*03 genes, it is well acknowledged that about 90–95% of CD patients are carryingDQ2.5 heterodimers encoded by DQB1*02 associated to DQA1*05 alleles in cis or in trans configuration, and DQ8 molecules withDQB1*03:02 generally combined to DQA1*03 variant [22–24]. However, HLA-DQ2 and HLA-DQ8 heterodimers were reported to explain only about 40% of CD heritability [25, 26]. The remaining 60% of genetic susceptibility to the disease were estimated to be shared between an unknown number of HLA and non-HLA genes [27, 28]. Although limited, the drawn groups (50 CD patients and 50 controls) may be considered as representative of the Mauritanian population (4.5 million inhabitants [29] with a (target population/general population) ratio quite above that reported in similar studies. For instance, DQB1/DRB1 PCR-based HLA typing was used on 143 Scandinavian CD patients and 135 non-celiac disease controls, out of a general population estimated to 28 million inhabitants [30]. In North Africa, similar approach was conducted on 40 CD patients and 40 Tunisians control from Tunisia with a total population of 12 million [31, 32].

The mechanism by which HLA alleles from various loci were involved in CD onset remained unclear. However, gluten proteins have been shown to contain various immunogenic peptides [33, 34]. Bearing in mind the striking polymorphism of HLA system and its specific antigen presenting function, a correlation between the numerous gluten epitopes and various corresponding HLA antigens competent of triggering a T cell reaction in celiac disease is readily reasonable. This association has been supported, for instance, by the difference in the level of gluten peptide binding to HLA-DQ2 homozygous and heterozygous APC [35, 36]. In this quantitative model, the relationship between HLA-DQB1 and DRB1 allele expression level and the available number gluten peptides may therefore be a critical limiting factor in the disease development [37]. According to this hypothesis, the non-association between CD and HLA-DQ2.2 [38, 39], in contrast to the significant risk by HLA-DQ2.5,would be the

result of a limited number HLA specific T cell response due to low amount of specific immunogenic gluten peptides [40, 41]. This HLA-gene dose effect induced by antigenic gluten epitopes could also be involved in other auto immune diseases such as type I diabetes, rheumatoid arthritis, or systemic lupus erythematosus [42, 43]. Although this study showed that, HLA-DQB1, HLA-DRB1 alleles and haplotypes conferred a significant risk of celiac disease in the Mauritanian population as in other populations worldwide, the disease susceptibility remained primarily mediated by HLA-DQ2 and DQ8 heterodimers which require both DQA1 and DQB1 alleles. Therefore, the present analysis provides only a partial characterization of the disease genetic risk. Future studies incorporating full HLA haplotyping including DQA1 locus are planned in order to substantially improve the clinical and genetic relevance of the findings.

## Conclusion

This study confirmed the contribution of bothHLA-DQB1*02 and HLA-DRB1*03 alleles and haplotypes in CD development in our population following a quantitative HLA-gene dose effect model.

Cross validation of the PCR–SSP HLA typing by NGS Nanopore sequencing technology supported the obtained data reliability. Besides, it allowed the availability of a simpler and affordable approach for genetic disorders investigation including celiac disease in which symptoms ambiguity during diagnosis is common.

However, Although HLA typing is useful in CD exclusion among individuals with equivocal clinical and laboratory findings, the presence of this at-risk genotype does not confer a diagnosis of the disease giving its low predictive value in the general population,

### Study limitations

One significant limitation of this work is that although we used a number of patients and controls in the order of similar studies reported, this size is relatively small for a genetic association study. It may for instance affect the stability of the odds ratios. We therefore acknowledge that these results ought to be considered with appropriate caution. A larger cohort is thus recommended to confirm these data.

Besides, in this study some children did not undergo an intestinal biopsy to confirm the diagnosis of celiac disease.

Although both CD cases and controls groups included subjects from different age, sex and ethnicity accurate matching for these factors was not achieved. We acknowledge the significant influence of this drawback on data analysis.

## Data Availability

The sequence data for the 24 patients typed by NGS using Nanopore technology (MinION) in our study are available as accessible links in a supplementary file.
